# Functionality, muscular strength and cardiorespiratory capacity in the elderly: relationships between functional and physical tests according to sex and age

**DOI:** 10.3389/fphys.2024.1347093

**Published:** 2024-03-01

**Authors:** Víctor Toro-Román, Pau Ferrer-Ramos, Víctor Illera-Domínguez, Carla Pérez-Chirinos, Bruno Fernández-Valdés

**Affiliations:** Department of Health Sciences, TecnoCampus, Research Group in Technology Applied to High Performance and Health, Universitat Pompeu Fabra, Mataró, Barcelona, Spain

**Keywords:** elderly, assessment, physical fitness, short physical performance battery, functional test

## Abstract

**Introduction:** There are several tests that provide information about physical fitness and functionality in older adults. The aims of this study were: (i) to analyze the differences between sex and age in functional, strength and cardiorespiratory tests; and (ii) to study the correlations between functional, strength and cardiorespiratory tests according to sex and age.

**Methods:** A total of 171 older adults (72.09 ± 13.27 kg; 1.59 ± 0.09 m; 72.72 ± 6.05 years) were divided according to sex (men: *n* = 63; women: *n* = 108) and age (≥60 <70: *n* = 65; ≥70 <80: *n* = 89; ≥80: *n* = 18). Anthropometry, body composition, upper limb strength (hand grip; HG), lower limb strength (countermovement jump; CMJ), cardiorespiratory capacity (6 min walking test; 6MWT), timed up and go test (TUG) and Short Physical Performance Battery (SPPB) were assessed.

**Results:** Men showed higher values in CMJ height, HG and expired volume (VE) (*p* < 0.05). There were no significant differences between sexes in TUG and SPPB. Regarding age, there were significant differences in CMJ, VE and peak oxygen uptake (VO_2peak_), TUG, gait speed, chair and stand test and SPPB total (*p* < 0.05). The test times were higher in older people. Regarding correlations, the TUG showed significant correlations in all strength and cardiorespiratory tests, regardless of sex and age. The CMJ correlated more significantly with functional tests compared to HG.

**Discussion:** There were sex and age differences in functional, strength, and cardiorespiratory tests. The execution of quick and low-cost tests such as the CMJ and TUG could provide information on overall physical fitness in older adults.

## 1 Introduction

Life expectancy is increasing world-wide, and it is estimated that by 2050, adults over 60 years of age will represent 21.1% of the world population (Nied and Franklin, 2002). During the same period, life expectancy is expected to increase, reaching 83 and 75 years in the most developed and least developed countries, respectively (Chatterji et al., 2015). However, following the COVID-19 pandemic, global life expectancy from 72.8 years in 2019 to 71.0 years in 2021, with an annual decrease of 1.2% (Cao et al., 2023).

Aging is the result of the accumulation of molecular and cellular degeneration over time, causing a gradual decline in physical and mental health (World Health Organization, 2015). The World Health Organization (WHO) defined “good health” as a state of complete physical, mental, and social wellbeing and not simply the absence of disease or illness (Frangos et al., 2023). Likewise, in 2015, the WHO defined the concept of “healthy aging” as the notion of functional capacity, rather than the mere presence or absence of disease. Being functional is the ability of an individual to live, be active, and take care of themselves independently (Fried et al., 2004). Functionality can be assessed through different methods, using a physical and cognitive approach.

During aging, the body undergoes a series of structural and functional changes in the different physiological systems. In general, progressive aging is associated with biochemical and functional changes in the cells of the musculoskeletal tissue, including the loss of strength and muscle mass, especially type II fibers, and the deterioration of the muscle’s oxidative capacity (Harber et al., 2009; Konopka and Harber, 2014). In addition, changes in the cardiovascular and respiratory system result in lower cardiac output and higher blood pressure, causing significant changes in the structure and function of the heart, as well as an alteration of oxygenation and a decrease in ventilation (Lakatta and Levy, 2003; Vigorito and Giallauria, 2014; Alvis and Hughes, 2015).

Personal autonomy depends on the individual’s ability to perform basic daily life activities. Loss of physical function leads to the onset of dependency and disability (Patrizio et al., 2021). Functional capacity and physical fitness are linked. Therefore, fitness assessment is becoming increasingly common (Cruz-Jentoft et al., 2010). The ability to maintain high levels of functioning in old age has been linked to the preservation of skeletal muscle function (Abreu et al., 2023). Decreased muscle strength and aerobic capacity is associated with decreased functional capacity and independence (Cruz-Jentoft et al., 2010).

In terms of physical assessment, there are several instruments and tests that report on the physical fitness and functionality of older adults. Among the most commonly used tests are the hand grip (HG), timed up and go test (TUG) and Short Physical Performance Battery (SPPB) (Guralnik et al., 1994; Mijnarends et al., 2013). The above tests are valid and reliable (Mijnarends et al., 2013). Another test widely used in the assessment of athletic performance is the countermovement jump (CMJ). However, in the assessment of older adults it is less common. Previous research suggested that the vertical jump action was more sensitive to age-related declines in neuromuscular function compared to conventional tests (Rittweger et al., 2001; Runge et al., 2004). It has been reported that vertical jump test performance procedures do not cause injury or increases in pain (Buehring et al., 2010).

Studies analyzing relationships between physical and functional tests in an elderly population have previously been published (Singh et al., 2014; Orssatto et al., 2020; Parsons et al., 2020; Staples et al., 2020; Santos et al., 2022; Abreu et al., 2023). However, few studies differentiate between sexes and age ranges (Buehring et al., 2015; Okabe et al., 2021; Monjo et al., 2023). It has been shown that muscle mass loss is twice as fast in men compared to women (Gallagher et al., 1997). Similarly, women have lower levels of physical fitness (Danneskiold-Samsøe et al., 2009) and experience a more rapid decline in physical performance (Samson et al., 2000). The prevalence of limitations in physical functioning correlates positively with age in both men and women (Okabe et al., 2021). However, information on the influence of sex and age is still scarce. On the other hand, it is known that power and muscle strength are related to measures of muscle function assessed by test batteries such as the SPPB (Parsons et al., 2020; Abreu et al., 2023). However, these relationships might be different between sexes and ages due to differences in muscle degeneration. Therefore, the aims of the present study were: i) to analyze the differences between sex and age range in functional, strength and cardiorespiratory tests; and ii) to study the correlations between functional, strength and cardiorespiratory tests as a function of sex and age range.

## 2 Material and methods

### 2.1 Participants

A total of 171 older subjects (72.09 ± 13.27 kg; 1.59 ± 0.09 m; 72.72 ± 6.05 years) divided into men (*n* = 63; 80.47 ± 11.27 kg; 1.68 ± 0.08 m; 72.84 ± 5.53 years) and women (*n* = 108; 67.34 ± 11.91 kg; 1.54 ± 0.06 m; 72.60 ± 6.36 years) participated in the present study. Similarly, participants were divided according to age: ≥60 <70 (*n* = 65; 73.25 ± 14.41 kg; 1.61 ± 0.09 m; 66.67 ± 2.18 years), ≥70 <80 (*n* = 89; 72.28 ± 12.83 kg; 1.59 ± 0.09 m; 74.84 ± 2.73 years) and ≥80 (*n* = 18; 67.00 ± 9.58 kg; 1.54 ± 0.10 m; 67.00 ± 9.58 kg 84.05 ± 3.57 years). Age ranges were established in line with previous authors (Milanović et al., 2013). All participants were verbally informed about the details of the study and gave written informed consent to participate in it. All procedures were approved by the university research ethics committee of TecnoCampus (Universitat Pompeu Fabra) (approval number: CEI1/2022) and were conducted in accordance with the Declaration of Helsinki.

Participants were recruited through digital channels, press and billboards in the Maresme region (Barcelona, Spain). The current sample is representative for an elderly population (over or equal to 60 years of age) in the province of Barcelona totaling 5609350 (according to data from the International Center on Aging) with a confidence level of 80% and a margin of error of 5%. To participate in the study, it was necessary to meet the following criteria: i) to reside in the Maresme region (Barcelona, Spain); ii) to have no medical contraindication for physical exercise; iii) to perform the physical fitness assessment tests without the need for assistance (personal or material); iv) to be over 60 years of age; and v) to give written consent. The study was carried out in different civic centers in Mataró.

### 2.2 Study design

This cross-sectional, descriptive, and quasi-experimental study lasted approximately 6 weeks, during the months of June and July 2023. Strength, cardiorespiratory and functional tests were performed. All assessments were carried out in the morning. Prior to the assessment, participants performed a 7-min warm-up that included walking at their own pace and upper and lower body joint mobility. The order of the assessments was as follows: first, anthropometry and body composition evaluations. The tests were then performed in the following order (Weakley et al., 2022): i) balance test; ii) HG; iii) CMJ; iv) gait speed; v) TUG; vi) Chair and stand test; and vii) 6 min walking test (6MWT). There was a 3-min rest period between all tests except between tests (vi) and (vii) where there was a 5-min rest period. It should be noted that the participants were not familiar with the tests performed. During the tests, three exercise professionals always supervised and monitored the safety of the study participants. Prior to the study, the raters were trained in the assessments used.

### 2.3 Anthropometry and body composition

Body height was assessed with a wall-mounted stadiometer (Seca 220; Hamburg, Germany). Body weight, fat-free mass and fat mass were estimated by electrical bioimpedance (Tanita, MC 780-P MA; Tokyo, Japan). Participants were assessed barefoot and with as little clothing as possible. Also, 3 days before the assessments, participants were instructed not to drink alcohol 48 h beforehand, to perform intense exercise at least 12 h beforehand and not to wear metallic objects.

### 2.4 Cardiorespiratory assessment

Cardiorespiratory fitness was assessed using the 6-min walking test (6MWT) (Bautmans et al., 2004). The test was performed outdoors on a hardened, flat surface following a 30 m straight line circuit. Participants were asked to try to cover as long a distance as possible in 6 minutes without running. Subjects wore comfortable clothing and footwear and were allowed to rest or stop when necessary. Participants were equipped with a portable gas analyzer (K5 COSMED; Rome, Italy) in order to assess ventilatory parameters objectively (Perez-Suarez et al., 2018). Peak oxygen uptake (VO_2peak_) and expired volume (VE), obtained in the 6MWT, were assessed.

### 2.6 Strength assessment

Upper body strength and lower body power were assessed using the manual grip test and the CMJ jump, respectively. Manual grip strength was measured with a Takei 5101 dynamometer (Takei Instruments Ltd., Tokyo, Japan). Participants performed two attempts with each hand with 30 s recovery and the best one was selected for analysis. Participants were encouraged to perform a maximal contraction while seated in a chair with the elbow flexed and the arm resting on the armrest. The grip piece of the dynamometer was adapted to the participants’ hands (Ortega et al., 2011). The CMJ test (Bosco et al., 1982) was used to assess lower body power. This type of test can be performed safely, without falls or accidents occurring during the test (Santos et al., 2022). The force platform (MuscleLab, Stathelle, Norway) was used to measure take-off height, power, and contact time (CT). Participants started the jump from an upright position, with feet shoulder-width apart and hands resting on the hips. Subjects performed a knee flexion-extension followed by a jump of maximum possible intensity. Two attempts were allowed with a 30 s rest period between jumps. The best jump was chosen for further analysis.

### 2.7 Functional test

The SPPB and the TUG test were used to assess the functional capacity of the study participants (Guralnik et al., 1994). A hand-held stopwatch (CASIO HS-30W-N1V, Tokyo, Japan) was used to monitor the tests. A videotape was made with detailed instructions for administering and scoring the functional tests, as well as instructions for keeping the subjects safe. Scores were assigned by the same rater. Several demonstrations were given by the rater prior to each test.

The TUG test is reliable, cost-effective, safe and efficient for assessing general functional mobility (Podsiadlo and Richardson, 1991; Kear et al., 2017). For the test, a chair with a backrest, an adhesive tape, and a cone to delimit the course were used. To perform the TUG test, subjects had to get up from the chair, walk 3 m, turn around, return to the chair, and sit down (Kear et al., 2017). Participants started the test seated, with their back against the backrest and their hands resting on their legs. The test time started with the word “go” (after a 3-s countdown) and ended when the participant was seated.

The standing balance tests were divided into three positions (de Fátima Ribeiro Silva et al., 2021):

1. Standing with feet side to side (at the same height) for 10 s.
2. Semi-tandem stance (one foot in front of the other foot touching, from the side, the heel of the forward foot to the big toe of the back foot) for 10 s.
3. Tandem stance (foot fully forward, with the heel of the forward foot in contact with the toes of the back foot) for 10 s.

For position one and two, the score was 0-1 according to the time the position was held (0 points: <10 s; 1 point: ≥10 s). For position three, the score was 0-1-2 (0 points: <3 s; 1 point: >3 <10 s; 2 points: ≥10 s. The total score for this test ranged from 0 to 4 points. For each position, the rater first demonstrated the task, then supported one arm while participants placed their feet, asked if they were ready, then released the support and began timing.

To assess walking speed, the 8-m course (2 m acceleration, 4 m time trial and 2 m deceleration, to avoid early braking bias) was marked out with no obstacles. Participants were instructed to walk at their usual speed, as if they were walking in the street. The timing of the test started with the word “go” (after a 3-s countdown) and ended when the participant passed the 4-m mark. Scoring was performed according to the following classification (0 points: the subject does not perform the test; 1 point: >6.52 s; 2 points: ≥4.66 ≤6.52 s; 3 points: ≥3.62 <4.65 s; and 4 points: <3.62 s (Paineiras-Domingos et al., 2018).

To assess the ability to stand up and sit down from the chair, a straight-backed chair was placed next to a wall. Participants were asked to cross their arms over their chest and stand up and sit down on the chair five times as quickly as possible. The time from the initial sitting position to the final standing position at the end of the fifth repetition was recorded. The score depends on the time of execution (0 points: ≥60 s; 1 point: ≥16.70 s; 2 points: ≥13.70 <16.70 s; 3 points: ≥11.20 <13.70 s; and 4 points: <11.20 s (Paineiras-Domingos et al., 2018).

### 2.7 Statistical analysis

Data are shown as mean ± standard deviation. Data were processed with IBM SPSS 25.0 Statistics (IBM Corp., Armonk, NY, United States) and R (v4.1.2, R Foundation for Statistical Computing, Vienna, Austria). The figures were created using the R commander program (v4.1.2, R Foundation for Statistical Computing, Vienna, Austria): The normality of the distribution of the variables was analyzed using the Kolmogorov-Smirnov test and the homogeneity of variances using the Levene test. A two-way ANOVA (effect of sex and effect age) was used to show any differences in the variables studied, as well as the interaction between the two effects. For this purpose, sex was coded on the one hand and age range on the other hand in order to separate the two categories. The sex effect refers to the differences between sexes in the parameters analysed and the age effect refers to the differences between the age ranges of the variables analysed. Finally, the sex × age interaction indicates whether the effects are dependent. The Bonferroni *post hoc* test was used to determined differences in effect age. Effect size was calculated using partial eta-squared: ≥0.01 <0.06 was a small effect size, ≥0.06 <0.14 moderate, and ≥0.14 large (Hopkins et al., 2009). Pearson’s correlation analysis and the simple linear regression model between tests were used. The magnitude of correlation was rated as trivial (<0.1), small (≥0.1 ≤0.29), moderate (>0.29 ≤0.49), large (>0.49 ≤0.69), very large (>0.69 ≤0.89) or nearly perfect (>0.9 ≤0.99) (Hopkins, 2002). A *p*-value <0.05 was considered statistically significant.

## 3 Results

The results obtained in the present study are shown below. Table 1 shows the data obtained in anthropometry and body composition. There were significant differences between sexes in height, weight, fat mass and fat free mass (*p* < 0.02). Men were taller, heavier and had higher fat free mass. Regarding age differences, significant differences were reported in height, fat mass and fat free mass (*p* < 0.02). Height and fat free mass decreased as a function of age. Large effect sizes were reported for height (sex) and moderate effect sizes for height (age), weight and fat free mass.

**TABLE 1:** Anthropometry and body composition.

Parameters	Age	Men	Women	Sex effect	Age effect	Sex × age
Height (m)	≥60 <70	1.71 ± 0.07**	1.57 ± 0.06**	<0.001##	<0.001#	0.037
	≥70 <80	1.69 ± 0.07**	1.52 ± 0.05*			
	≥80	1.54 ± 0.03	1.51 ± 0.08			
Weight (kg)	≥60 <70	82.24 ± 7.51	67.38 ± 12.86	<0.001#	0.409	0.876
	≥70 <80	79.33 ± 12.00	66.45 ± 11.52			
	≥80	74.80 ± 9.33	63.32 ± 7.89			
BMI	≥60 <70	28.28 ± 3.20	27.20 ± 5.15	0.302	0.589	0.334
	≥70 <80	27.72 ± 3.44	28.41 ± 4.06			
	≥80	31.27 ± 2.47	27.84 ± 5.08			
Fat mass (%)	≥60 <70	23.88 ± 5.94*	30.66 ± 7.95*	0.017	0.018	0.378
	≥70 <80	25.78 ± 4.83	33.65 ± 7.49			
	≥80	34.95 ± 7.14	34.90 ± 8.22			
Fat free mass (%)	≥60 <70	75.46 ± 6.74*	68.67 ± 7.66*	0.007	0.013#	0.288
	≥70 <80	73.74 ± 5.12	65.36 ± 5.56			
	≥80	64.05 ± 7.14	64.79 ± 8.50			

*p < 0.05; **p < 0.01 differences vs. ≥80; #: moderate effect size; ##: large effect size; BMI: body mass index.

Table 2 shows the results obtained in the strength and cardiorespiratory assessment tests. There were significant differences between sexes in CMJ height, HG and VE (*p* < 0.05), being higher in men. With respect to age, significant differences were observed in CMJ, VE, and VO_2peak_, with lower values in older participants (*p* < 0.05).

**TABLE 2:** Strength and cardiorespiratory assessment.

Parameters	Age	Men	Women	Sex effect	Age effect	Sex × age
CMJ Height (cm)	≥60 <70	14.09 ± 6.71^^	12.32 ± 2.56^^	0.025	<0.001#	0.218
	≥70 <80	12.94 ± 3.65++	9.01 ± 4.31++			
	≥80	7.72 ± 4.14++^^	7.09 ± 3.98++^^			
CMJ CT (ms)	≥60 <70	221.8 ± 140.8**	210 ± 2±108.4**	0.237	0.041	0.756
	≥70 <80	236.0 ± 102.3*	196.0 ± 83.7*			
	≥80	153.6 ± 87.7	122.8 ± 89.7			
CMJ (W/kg)	≥60 <70	10.61 ± 5.29+	7.94 ± 3.56+	0.036	<0.001#	0.503
	≥70 <80	8.56 ± 4.36++	6.03 ± 2.64++			
	≥80	4.70 ± 2.40	4.66 ± 2.15			
HG Right (kg)	≥60 <70	32.98 ± 12.48	20.65 ± 8.65	<0.001##	0.106	0.849
	≥70 <80	29.85 ± 11.31	22.69 ± 10.55			
	≥80	26.10 ± 9.07	20.53 ± 8.86			
HG left (kg)	≥60 <70	31.43 ± 11.43	18.70 ± 8.83	<0.001##	0.176	0.440
	≥70 <80	29.77 ± 9.65	14.46 ± 6.83			
	≥80	27.70 ± 6.76	18.31 ± 2.40			
VE (L/min)	≥60 <70	64.91 ± 22.61	40.56 ± 13.52	<0.001#	<0.001##	0.106
	≥70 <80	47.80 ± 16.76++	32.98 ± 14.53++			
	≥80	35.03 ± 13.88++	30.08 ± 16.92++			
VO2peak (mL/kg/min)	≥60 <70	24.30 ± 11.17	23.46 ± 10.15	0.762	0.005	0.479
	≥70 <80	22.19 ± 5.30+	18.87 ± 8.02+			
	≥80	14.05 ± 10.14++	16.44 ± 8.51++			

+*p* < 0.05; ++*p* < 0.01; differences ≥60 < 70; *p < 0.05; **p < 0.01 differences vs. ≥80; ^*p* < 0.05; ^^*p* < 0.01 differences vs. ≥70 < 80; #: moderate effect size; ##: large effect size; CMJ: countermovement jump; CT: contact time; HG: hand grip; VE: expired volume; VO_2peak_: peak oxygen uptake.

Table 3 reports the results obtained in the functional tests (SPPB and TUG) evaluated in the present study. No significant differences between sexes were reported. Regarding age differences, there were significant differences in TUG, gait speed, semi-tandem stance, SPPB chair stand, SPPB gait time and total SPPB (*p* < 0.05). In older people, test times were higher and reported lower SPPB scores. Regarding sex × age interactions, there were significant differences in semi-tandem and tandem stance and total SPPB (*p* < 0.05). Finally, regarding effect sizes, there were moderate effect sizes in TUG, gait speed, SPPB chair stand test, SPPB gait speed and balance tests. In addition, the effect size in total SPPB was large in the age effect.

**TABLE 3:** Functional test.

Parameters	Age	Men	Women	Sex effect	Age effect	Sex × age
TUG (s)	≥60 <70	6.43 ± 1.72	6.71 ± 0.99	0.942	<0.001#	0.880
	≥70 <80	7.14 ± 1.55	7.21 ± 2.04			
	≥80	9.09 ± 2.56	8.83 ± 3.67			
Speed gait (m/s)	≥60 <70	1.12 ± 0.37**	1.19 ± 0.24**	0.978	0.001#	0.223
	≥70 <80	1.18 ± 0.33**	1.07 ± 0.34**			
	≥80	0.78 ± 0.03	0.82 ± 0.34			
Chair Stand (s)	≥60 <70	7.50 ± 3.43**	8.67 ± 2.68**	0.218	0.132	0.323
	≥70 <80	8.74 ± 4.45**	8.31 ± 3.90**			
	≥80	9.28 ± 5.04	11.63 ± 6.11			
Side-by Side Stand (point)	≥60 <70	1.00 ± 0.00	1.00 ± 0.00	1.000	1.000	1.000
	≥70 <80	1.00 ± 0.00	1.00 ± 0.00			
	≥80	1.00 ± 0.00	1.00 ± 0.00			
Semi tandem stand (point)	≥60 <70	1.00 ± 0.00	1.00 ± 0.00	0.057	0.038	0.006#
	≥70 <80	1.00 ± 0.00	0.95 ± 0.20			
	≥80	0.80 ± 0.44	1.00 ± 0.00			
Tandem stand (point)	≥60 <70	1.85 ± 0.48	1.90 ± 0.29	0.069	0.195	0.048#
	≥70 < 80	1.86 ± 0.42	1.81 ± 0.39			
	≥80	1.40 ± 0.89	1.92 ± 0.27			
SPPB Chair Stand (point)	≥60 <70	3.80 ± 0.52**	3.76 ± 0.52**	0.548	0.002#	0.880
	≥70 <80	3.72 ± 0.65**	3.70 ± 0.58**			
	≥80	3.20 ± 0.83	3.00 ± 1.15			
SPPB Gait time (point)	≥60 <70	3.90 ± 0.30**	3.90 ± 0.29**	0.813	<0.001#	0.854
	≥70 <80	3.75 ± 0.60	3.70 ± 0.45			
	≥80	3.20 ± 0.44	3.30 ± 0.85			
SPPB Total (point)	≥60 <70	11.55 ± 0.99**	11.58 ± 0.79**	0.451	<0.001##	0.048
	≥70 <80	11.33 ± 1.04**	11.18 ± 0.98**			
	≥80	9.60 ± 1.51	10.23 ± 2.00			

*p < 0.05; **p < 0.01 differences vs. ≥80; #: moderate effect size; ##: large effect size; TUG: timed up and go; SPPB: short physical performance battery.

**FIGURE 1:**
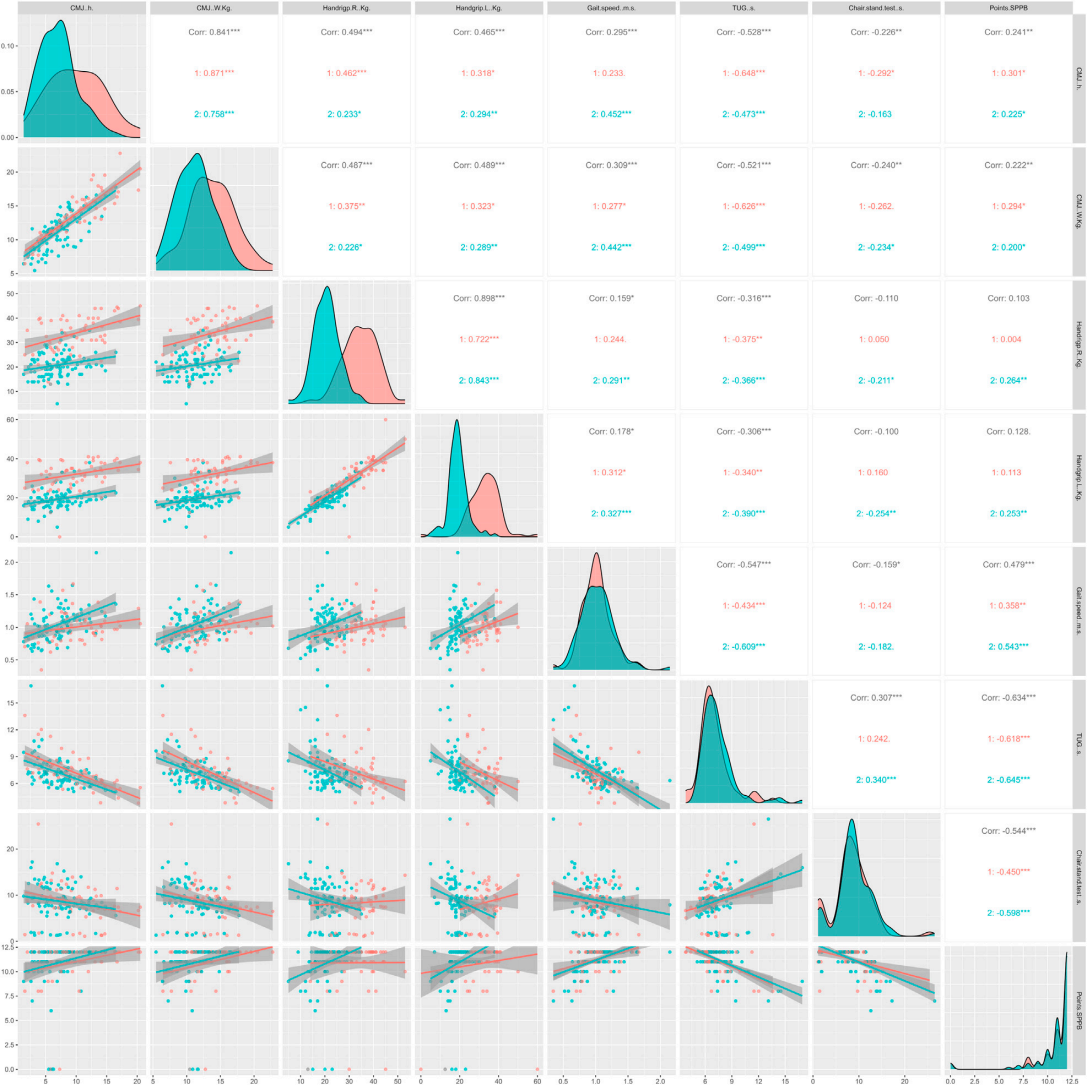
Regression lines, according to sex, between conventional strength tests and functional tests; 1 = men; 2 = women; Corr = overall correlation; *p < 0.05; **p < 0.01; ***p < 0.001 in the correlations analyzed; CMJ, countermovement jump; TUG, timed up and go; SPPB, Short Physical Performance Battery.

**FIGURE 2:**
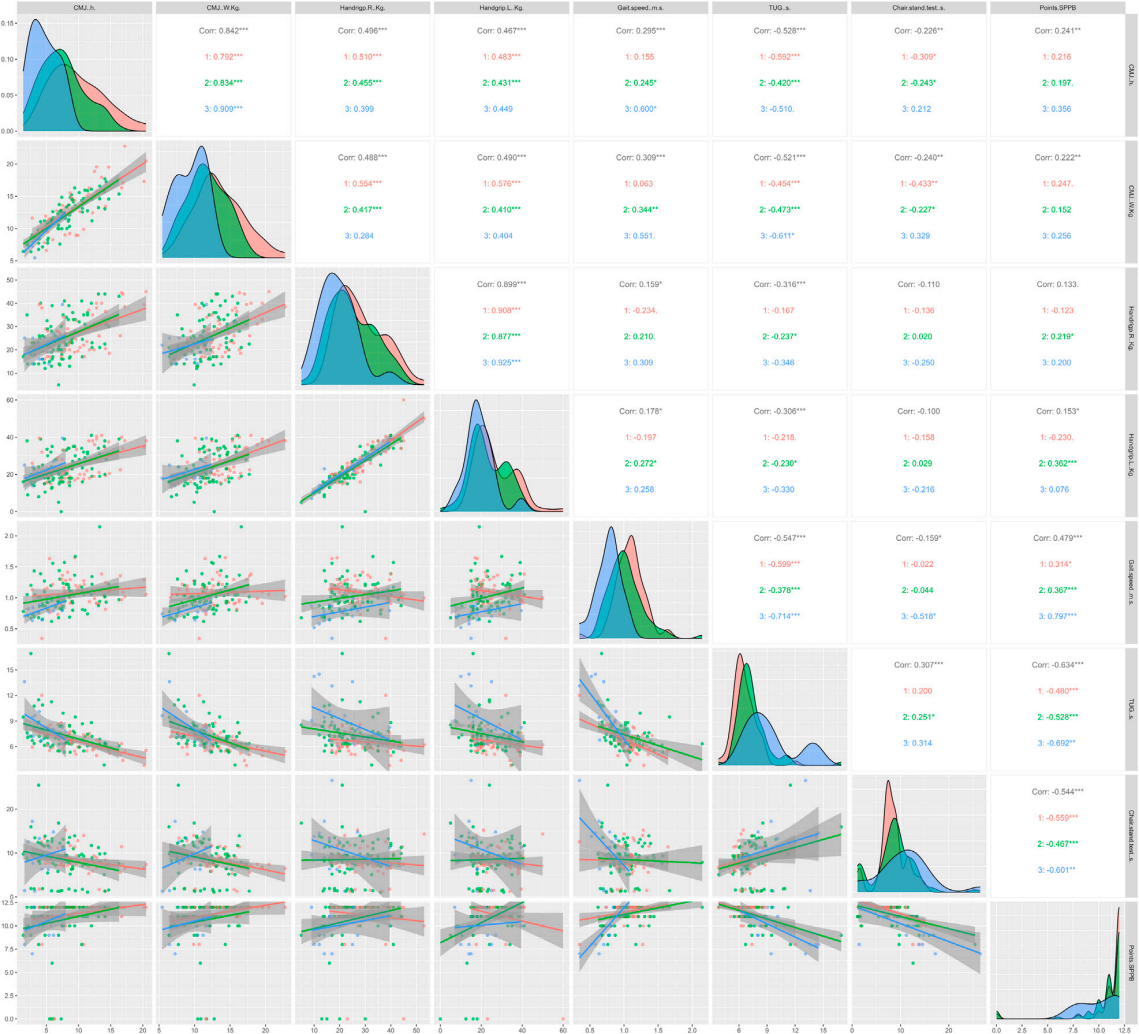
Regression lines, according to age, between conventional strength tests and functional tests; 1 = ≥60 <70 years; 2 = ≥70 <80 years; 3 = ≥80 years; Corr = overall correlation; *p < 0.05; **p < 0.01; ***p < 0.001 in the correlations analyzed; CMJ, countermovement jump; TUG, timed up and go; SPPB, Short Physical Performance Battery.

The figures below show the regression lines as a function of age and sex for the different tests used. Figure 1 shows the regression lines according to sex between the conventional strength tests and the functional tests. On the other hand, Figure 2 shows the regression lines for the same tests as a function of age.

Overall, significant correlations (*p* < 0.05) were observed between all strength and functional tests except HG with the SPPB score and chair stand test. In Figure 1, TUG showed strong significant correlations in all strength tests, independent of sex. In addition, the CMJ seems to correlate more strongly with the rest of the functional tests, being higher in men compared to women. Regarding Figure 2, TUG presented very significant correlations compared to the rest of the functional tests (*p* < 0.05), as in Figure 1. Similarly, CMJ correlated more strongly with the functional tests compared to HG. The age group with the highest significant correlations was ≥70 <80 years.

**FIGURE 3:**
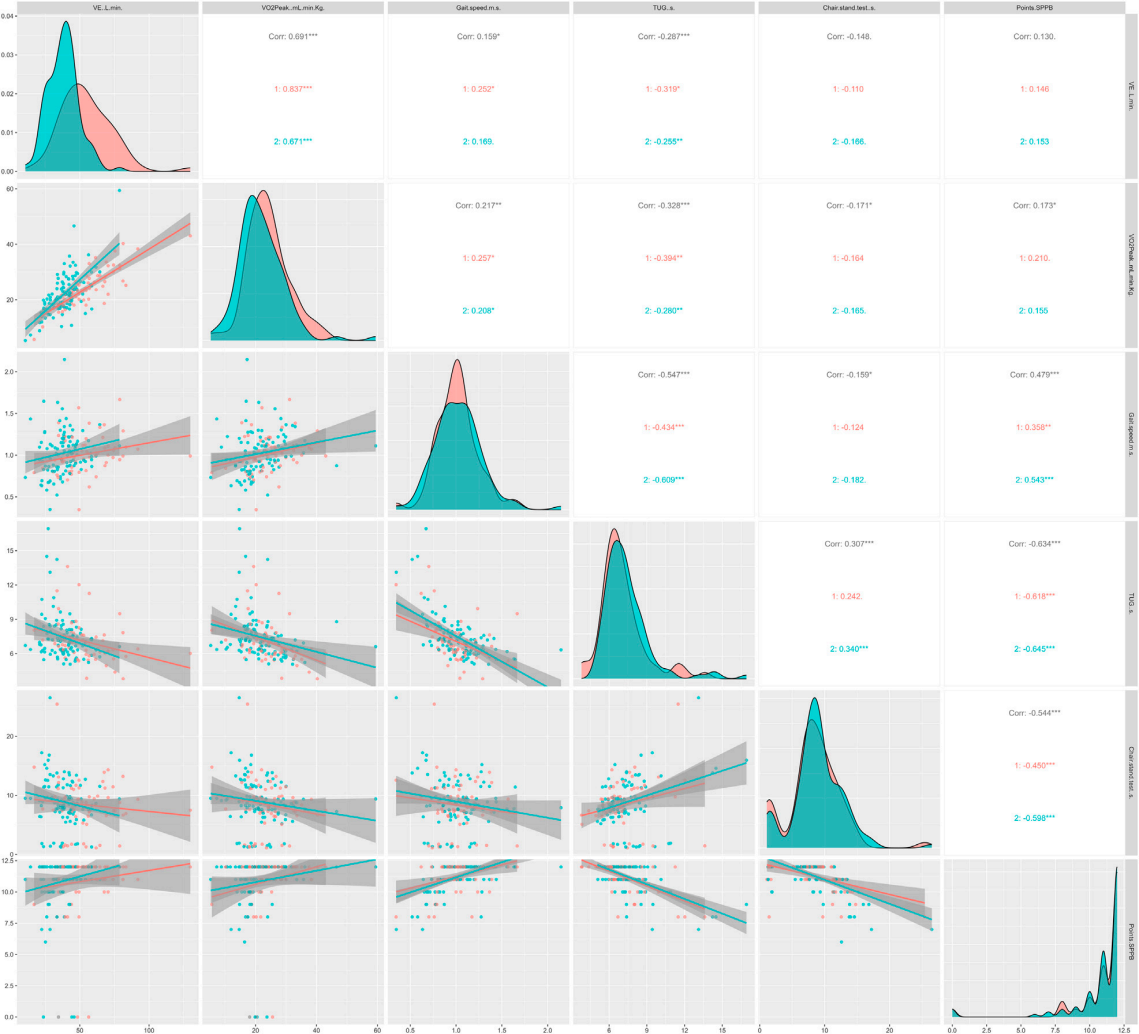
Regression lines, according to sex, between conventional endurance test and functional tests; 1 = men; 2 = women; Corr = overall correlation; *p < 0.05; **p < 0.01; ***p < 0.001 in the correlations analyzed; VE: expired volume; VO_2peak_: peak oxygen uptake; TUG: timed up and go; SPPB: Short Physical Performance Battery.

**FIGURE 4:**
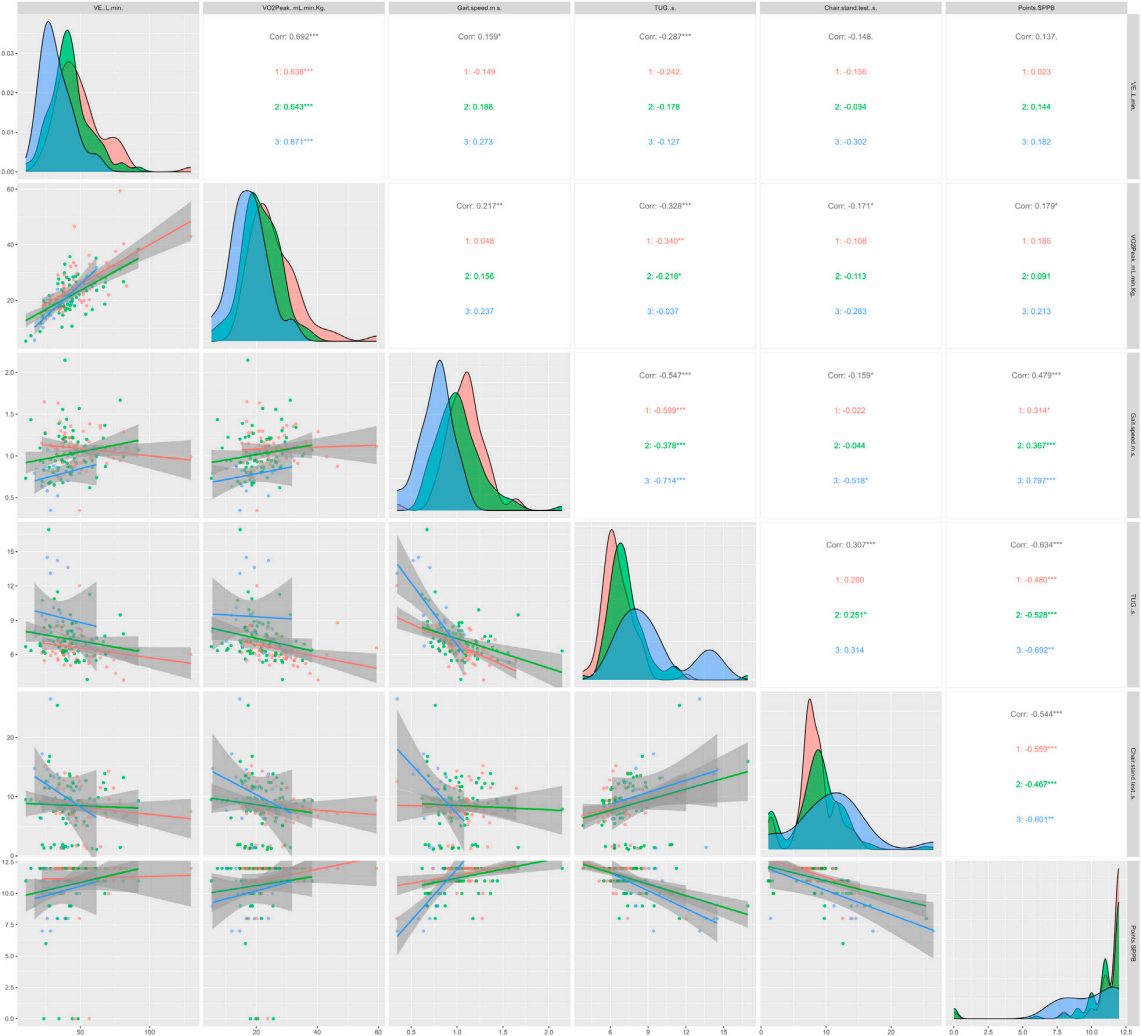
Regression lines, according to age, between conventional endurance test and functional tests; 1 = ≥60 <70 years; 2 = ≥70 <80 years; 3 = ≥80 years; Corr = overall correlation; *p < 0.05; **p < 0.01; ***p < 0.001 in the correlations analyzed; VE, expired volume; VO_2peak_, peak oxygen uptake; TUG, timed up and go; SPPB, Short Physical Performance Battery.

Figures 3, 4 show the regression lines and correlations between cardiorespiratory and functional test as a function of sex (Figure 3) and age (Figure 4). In Figure 3, in general, high correlations were observed between all the tests analyzed. However, with chair stand tests the correlations were low. As in the strength tests, TUG obtained highly significant correlations with the cardiorespiratory parameters analyzed, being higher in men (*p* < 0.05). In terms of age differences, TUG was the test that correlated best with cardiorespiratory parameters in general and in the ages analyzed as well. However, age range ≥80 years was the group that showed the lowest correlations.

**FIGURE 5:**
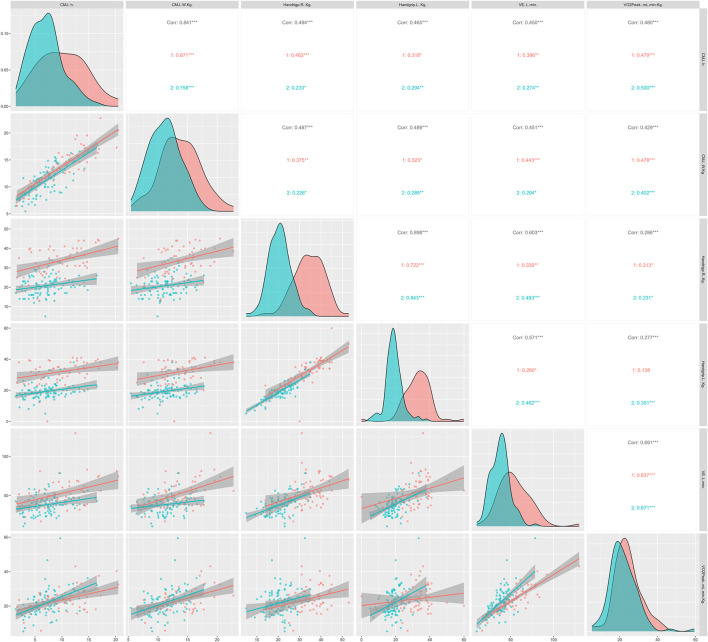
Regression lines, according to sex, between cardiorespiratory test and conventional strength tests; 1 = men; 2 = women; Corr = overall correlation; *p < 0.05; **p < 0.01; ***p < 0.001 in the correlations analyzed; CMJ, countermovement jump; HG, hand grip; VE, expired volume; VO_2peak_, peak oxygen uptake.

**FIGURE 6:**
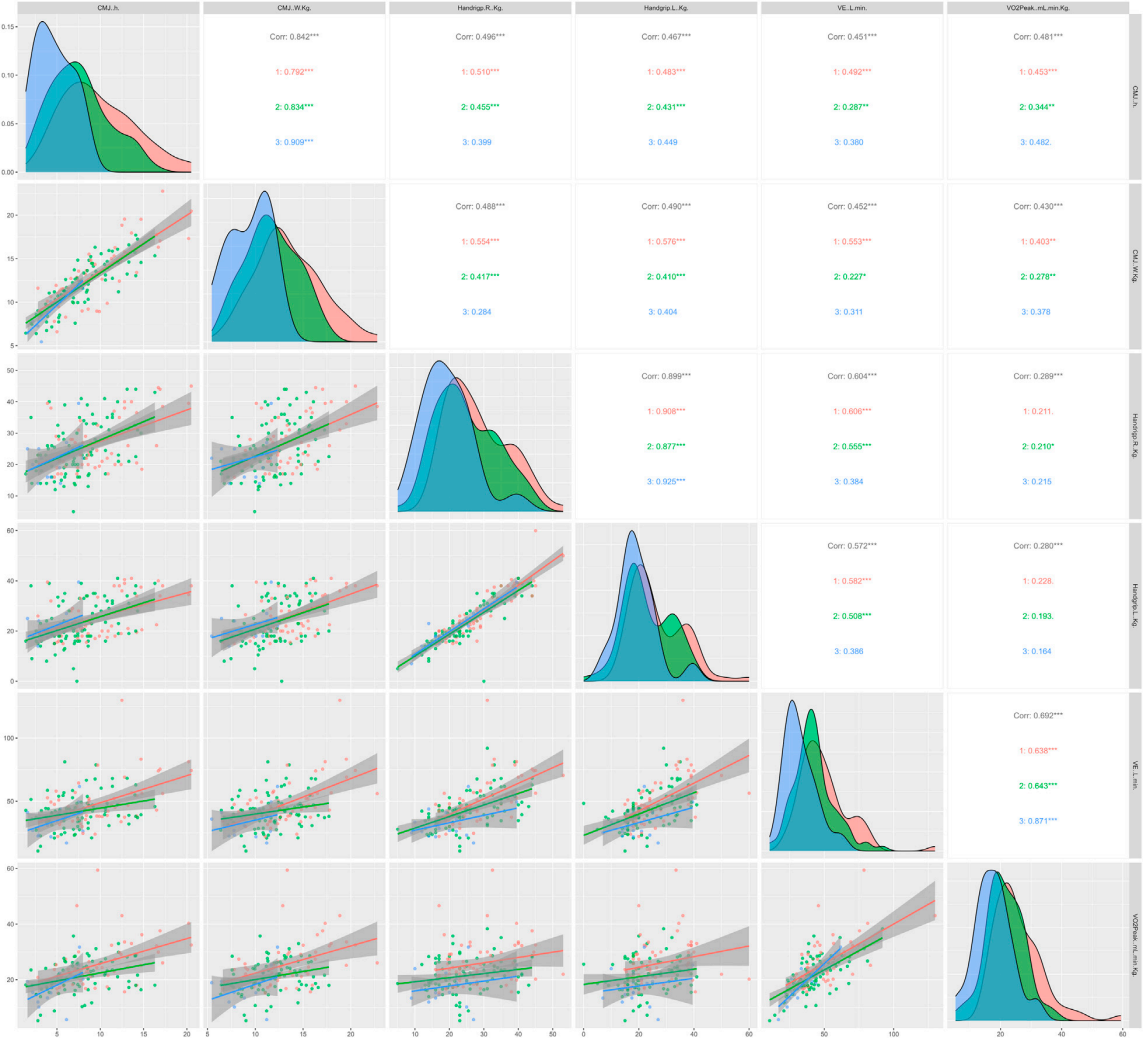
Regression lines, according to sex, between cardiorespiratory test and conventional strength tests; 1 = ≥60 <70 years; 2 = ≥70 <80 years; 3 = ≥80 years; Corr = overall correlation; *p < 0.05; **p < 0.01; ***p < 0.001 in the correlations analyzed; CMJ: countermovement jump; HG: hand grip; VE: expired volume; VO_2peak_: peak oxygen uptake.

Finally, Figures 5, 6 show the correlations between the strength and cardiorespiratory tests with the functional tests analyzed, according to sex (Figure 5) and age ranges (Figure 6). In Figure 5, CMJ correlated most significantly with cardiorespiratory parameters. The correlations in CMJ and HG with cardiorespiratory parameters were higher in men. In addition, HG correlated more strongly with VE compared to VO_2peak_. Similarly, in Figure 6 a similar trend to Figure 5 was maintained, CMJ showed a higher correlation with cardiorespiratory parameters compared to HG. The highest age range (≥80 years) showed a greater number of non-significant correlations.

## 4 Discussion

The aims of the present study were: i) to analyze differences between sex and age groups in functional tests, strength tests, and cardiorespiratory tests; and ii) to examine correlations between functional tests, strength tests, and cardiorespiratory tests based on sex and age. Significant sex differences were found in CMJ height, HG, and VE (*p* < 0.05), with men exhibiting higher values. However, no differences were observed in the functional tests. Concerning age, significant differences were noted in all analyzed parameters of CMJ, VE, and VO_2peak_, TUG, walking speed, semi-tandem stand, SPPB Chair Stand, SPPB gait time, and SPPB total (*p* < 0.05). Test times were higher in older individuals, resulting in lower total scores in SPPB for the elderly. Regarding correlations, TUG showed strong significant correlations with all strength and cardiorespiratory tests, regardless of sex and age. On the other hand, CMJ correlated more significantly with functional tests. These findings align with those observed by other authors who reported that CMJ is a highly reproducible and safe functional test, akin to HG (Buehring et al., 2010). However, HG is an isometric exercise that evaluates only one muscle group. Conversely, TUG is one of the most popular tools for assessing functionality in older adults (Podsiadlo and Richardson, 1991). Both TUG and CMJ require coordination and safely assess an individual’s maximum effort. Hence, they may have advantages over traditional functional/muscular tests in older adults, being rapid and cost-effective with validated low-cost measurement tools (Balsalobre-Fernández et al., 2015).

Regarding functional physical assessment in aging, it is well-established that various instruments and tests not only verify the ability of older adults to perform everyday tasks but also aid in identifying vulnerabilities for the development of frailty and sarcopenia (Mijnarends et al., 2013). It is conceivable that a single execution of some of these tests could provide comprehensive information due to their interrelation with the rest of the assessments.

It is well-known that the most widely used tests for assessing functional capacity and/or mobility in older adults include the TUG, 6MWT, and the Chair Stand Test (de Fátima Ribeiro Silva et al., 2021). The SPPB is one of the most employed batteries to assess the physical functioning of older individuals. Previous studies analyze the ceiling and floor effect of SPPB. However, it appears to be less suitable for assessing fitness level in healthy older adults or discriminating performance in severely disabled older people. When a measure is used to capture change, high baseline scores and ceiling effects limit the ability to detect improvement. Therefore, assessing SPPB performance over time may be more appropriate (Bergland and Strand, 2019). Longitudinal epidemiological studies have demonstrated its ability to predict dependence and mortality states (Studenski et al., 2003; Onder et al., 2005). Furthermore, a strong association may exist between SPPB and multiple measures of functional status (Cesari et al., 2006; Cabrero-García et al., 2012). Additionally, the TUG test is simple, quickly administered, and requires minimal equipment (Kear et al., 2017). Previous studies have reported that the SPPB score decreased by approximately 0.5 (0.17 points/year) over 3 years or 0.6 points/year (Sergi et al., 2011).

Concerning strength and cardiorespiratory tests, it has been demonstrated that HG is valid for assessing overall muscular strength (Bohannon, 2001; McGrath et al., 2018) and the risk of falls (Neri et al., 2021). Reduced levels of HG are associated with premature mortality and disability (McGrath et al., 2018). Specifically, individuals with low grip strength were significantly more likely to walk at ≤0.4 m/s (odds ratio 2.77), be unable to get up from a chair (odds ratio 2.73) and do heavy housework (odds ratio 1.69) (Rantanen et al., 1999). Similarly, vertical jump tests have been used to evaluate muscle function in individuals with reduced muscle functionality (Buehring et al., 2010; Buehring et al., 2015). Additionally, vertical jump tests appear to be safe even in advanced age (Runge et al., 2004; Buehring et al., 2010). However, their assessment might pose challenges in clinical settings. On the other hand, performance in the 6MWT is highly correlated with that of the 12-min walk test from which it was derived (Bautmans et al., 2004). The 6MWT is a valid tool for evaluating the progression of functional exercise capacity in clinical settings (Bautmans et al., 2004).

Anthropometric values and body composition (Milanović et al., 2013; Soh and Won, 2021), and the results of the strength and cardiorespiratory tests (Buehring et al., 2015; Okabe et al., 2021; Monjo et al., 2023), as well as the functional tests (Cabrero-García et al., 2012; Buehring et al., 2015; Okabe et al., 2021; Monjo et al., 2023), were similar to those reported by other authors. In relation to the above, significant inverse relationships have been reported between BMI and gait speed, as well as inverse relationships between body weight and the chair and stand test (Ramírez-Vélez et al., 2020).

Regarding sex differences, the results indicated that men were taller, heavier, and had a higher lean body mass. Additionally, the CMJ height, HG strength, and VE were also higher in men (*p* < 0.05). Previous authors reported similar data on body composition parameters in a population over 60 years old (Milanović et al., 2013; He et al., 2018). Concerning strength and cardiorespiratory tests, previous studies have reported gradual declines in HG strength in both sexes, with the most significant decline in women (Samson et al., 2000; Huebner et al., 2022). Regarding CMJ, differences between sexes have been observed in older age, in former athletes (Alvero-Cruz et al., 2021), and healthy adults (Siglinsky et al., 2015). Regarding VE, similar results have been reported in elderly individuals (Molgat-Seon et al., 2018).

The deterioration of muscle strength and function is a consequence of the aging process and may be associated with functional limitations, the risk of falls (Fukagawa et al., 1995), and a loss of bone mineral density (Sinaki et al., 1986). The reduction in related mobility and agility is more prevalent in women (Haynes et al., 2020). It is well-known that the loss of strength and muscle mass is a multifactorial process driven by hormonal alterations, nutritional factors, inflammation, and pathological states (Cruz-Jentoft et al., 2014; Tay et al., 2015). Some circulating hormones, such as insulin-like growth factor 1 (IGF-1) and testosterone, play important roles in regulating muscle mass as they participate in muscle protein synthesis (Yarasheski, 2003) and/or satellite cell activation (Roberts et al., 2018). Additionally, the decline in estrogen concentrations after menopause partially contributes to the decrease in muscle size and strength among women (Horstman et al., 2012). Regarding muscle fibers, some studies have suggested that men have a higher proportion of type II muscle fibers (Trevino et al., 2019; Haynes et al., 2020). This difference in fiber composition could contribute to distinctive rates of strength loss with age (Trevino et al., 2019). Concerning lung function, women have smaller lungs and lower maximum expiratory flows than men (Crapo et al., 1982). Moreover, women have smaller airways than men (Molgat-Seon et al., 2018). These factors could explain the sex differences observed in the tests used.

Previous cross-sectional studies have observed a similar trend in fat mass, increasing linearly with age (Bazzocchi et al., 2013). This trend was also noted in 2-year follow-up studies (Gallagher et al., 2000; Zamboni et al., 2003). Regarding vertical jump performance, previous studies have reported declines in CMJ height, being greater in individuals of very advanced age (Runge et al., 2004; Alvero-Cruz et al., 2021). Concerning VO_2peak_, decreases of approximately 10% per decade have been reported (Robinson, 1938; Hawkins and Wiswell, 2003). Additionally, other authors have observed an acceleration in the decline of VO_2peak_, ranging from 3% to 6% every 10 years in individuals aged 20 to 30, to 20% every 10 years starting from the age of 70 (Fleg et al., 2005).

It is well-known that fat mass progressively increases in both men and women throughout the life cycle. A high level of body fat is associated with poorer physical performance in older adults, and the accumulation of fat within skeletal muscles is linked to muscular weakness and impaired muscle function (He et al., 2018). The decline in muscle strength and power related to age could be attributed to various factors. Notable among these are increased fat mass (Baumgartner et al., 1998), decrease muscle mass and the cross-sectional area (Runge et al., 2004), fiber pennation angle impairment (Narici et al., 2003) and an altered hormonal environment in combination with systemic inflammation(Merritt et al., 2013; Roberts et al., 2018) among other factors. Systemic hormones such as IGF-1, growth hormone, and testosterone decrease with age (Vitale et al., 2016). In the early stages of aging, women lose muscle mass and strength more rapidly than men due to the reduction in estrogen secretion during menopause. However, in later stages, men experience declines in IGF-1 and testosterone levels, leading to a greater rate of muscle function decline and muscle mass loss (Morley et al., 2014).

Balance capacity is associated with muscular weakness (Alonso et al., 2018) and is considered a comprehensive index of physical function. The decline in age-related physical function occurs with a reduction, among other factors, in coordination and motor control. Changes in sensory receptors and peripheral nerves associated with decreased visual acuity and vestibular function affect postural control, leading to a decline in postural balance (Okabe et al., 2021).

Regarding the decline in VO_2peak_ during aging, there are central and peripheral factors that could explain this trend. Concerning central factors, maximum heart rate decreases by approximately 3%–5% per decade regardless of sex and training level (Hawkins and Wiswell, 2003). Similarly, stroke volume decreases, with a more pronounced effect in sedentary individuals. Consequently, cardiac output is also reduced (Hawkins and Wiswell, 2003). Additionally, it is believed that the primary change in the respiratory system related to aging is the reduction in lung elastic capacity (Molgat-Seon et al., 2018). Regarding peripheral factors, aside from changes in body composition, aging results in reductions in the arteriovenous oxygen difference, causing less oxygen utilization by skeletal muscles (Wiebe et al., 1999).

The correlation data revealed significant associations in all strength and cardiorespiratory tests in general with the TUG. When differentiating by sex, the correlations were higher in the men. On the other hand, the CMJ exhibited more significant correlations with functional and cardiorespiratory tests. Previous studies have observed significant correlations between relative power and maximum vertical jump velocity (Parsons et al., 2020; Abreu et al., 2023). Similar to the current study, previous work by Winger et al. reported that relative power in vertical jump and speed at maximum power showed associations twice as strong compared to HG strength (Winger et al., 2020). Concerning TUG, consistent with our study, it correlated significantly with squats, walking speed, SPPB, and the 30-s sit-to-stand test (Santos et al., 2022), exhibiting higher correlations compared to HG (Staples et al., 2020). Several studies emphasize that lower limb muscle power might be a more relevant marker for aging compared to HG and muscle mass (Runge et al., 2004; Parsons et al., 2020; Abreu et al., 2023). Power and speed could be more crucial factors in preventing functional loss in older individuals compared to muscle strength (Winger et al., 2020). Often, during aging, there is an observed loss of maximum strength. However, muscular power is the first component affected during the aging process (Cruz-Jentoft et al., 2010; Cruz-Jentoft et al., 2014). The age-related decline in the cross-sectional area of type II muscle fibers could explain the losses in muscle power and speed compared to strength (Winger et al., 2020).

The present study has certain limitations, including: i) the lack of consideration for medication intake, which could affect body composition and physical parameters, altering the values; ii) the absence of information on pre-existing pathologies; iii) the floor and ceiling effect was not analysed; iv) no familiarization test was performed, this could influence the results as the participant was not aware of the physical demands and the protocol; and v) the non-assessment of hydration status, which could potentially influence body composition values.

## 5 Conclusion

Differences were observed between sexes and age groups in functional, strength, and cardiorespiratory tests. Specifically, men exhibited a superior performance in cardiorespiratory and strength tests. Regarding age, scores and results in performance and functional tests decreased with increasing age of the subjects. The elderly individuals demonstrated diminished functional capacity. The CMJ and TUG tests appeared to have the strongest correlations with strength and cardiorespiratory tests, as well as functional tests, irrespective of age and sex.

Given the nature of the tests, the CMJ and TUG could provide comprehensive information about physical fitness and functionality in elderly individuals. Moreover, they are quick to administer, minimally invasive, and economically accessible for all professionals in the field of physical activity and sports sciences working with older populations.

## Data Availability

The raw data supporting the conclusion of this article will be made available by the authors, without undue reservation.
